# Draft Genome Sequence of Streptomyces spongiicola Strain 531S, an Actinobacterium Isolated from Marine Sediment

**DOI:** 10.1128/MRA.01198-18

**Published:** 2019-01-17

**Authors:** Hideo Dohra, Issara Kaweewan, Beatriz E. Casareto, Yoshimi Suzuki, Shinya Kodani

**Affiliations:** a Research Institute of Green Science and Technology, Shizuoka University, Suruga-ku, Shizuoka, Japan; b Graduate School of Integrated Science and Technology, Shizuoka University, Suruga-ku, Shizuoka, Japan; c Graduate School of Science and Technology, Shizuoka University, Suruga-ku, Shizuoka, Japan; University of Delaware

## Abstract

Streptomyces spongiicola strain 531S (NBRC 113560) was isolated from marine sediment on a beach on Sesoko Island (Okinawa, Japan). We report here the draft genome sequence of S. spongiicola 531S, in which 24 potential secondary metabolite gene clusters were predicted with antiSMASH.

## ANNOUNCEMENT

Streptomyces spongiicola (HNM0071^T^) was previously described as a novel actinomycete species (1). We isolated S. spongiicola strain 531S (NBRC 113560) from marine sediment on a beach on Sesoko Island (Okinawa, Japan) using International Streptomyces Project 3 (ISP3) (2) agar medium. To clarify its potential for biosynthesis of secondary metabolites, we determined the draft genome sequence of S. spongiicola 531S.

S. spongiicola 531S was cultured in ISP2 (2) medium, and the DNA was extracted with a DNeasy blood and tissue kit (Qiagen). A paired-end library of the genome was constructed as previously described (3) and sequenced with the Illumina MiSeq platform (302-bp paired end). The raw read sequences were cleaned with Trimmomatic (4) as described previously (3). The 3,210,816 high-quality reads totaling approximately 730 Mb were assembled with SPAdes v. 3.11.1 (5) with a parameter setting as described previously (3), and contigs less than 500 bp, in which no protein-coding sequence was predicted, were removed. The resulting 84 contigs showed a 105.6-fold coverage, and the *N*_50_ value was 395,703 bp.

The draft genome of S. spongiicola 531S has a total size of 6,912,677 bp with a G+C content of 72.61%. The average nucleotide identity (ANI) of the genome was calculated using the ani.rb script from the enveomics collection (6). The ANI analysis showed a high ANI value (99.0%) with the complete genome sequence of S. spongiicola HNM0071^T^ (accession no. CP029254). Gene prediction and annotation were performed using the DFAST-core stand-alone program v. 1.0.7 (7) with a S. spongiicola HNM0071^T^ protein database constructed with a utility script bundled in the DFAST-core and manually curated. The S. spongiicola 531S genome contained 5,874 protein-coding sequences, 81 tRNA genes, and 19 clustered regularly interspaced short palindromic repeat (CRISPR) sequences. Among 5,874 proteins, 5,003 (85.17%) were predicted to be orthologous proteins, with those of S. spongiicola HNM0071^T^ in reciprocal best matches using a blastp search (6) with the following settings: identity, >50%; E value, <1e-10; and query sequence coverage, >50%.

Secondary metabolite biosynthesis gene clusters of the S. spongiicola 531S genome were predicted with the antiSMASH 4.1.0 bacterial version (https://antismash.secondarymetabolites.org/) (8). The S. spongiicola 531S genome sequence contained 24 putative biosynthetic gene clusters, including 9 clusters involved in the biosynthesis of the following bioactive peptides: 3 bacteriocins, 2 thiopeptides, 1 nonribosomal peptide synthetase (NRPS), and hybrid clusters of a ladderane/arylpolyene/NRPS, a lantipeptide/type I polyketide synthase (PKS)/other type of PKS, and a type I PKS/butyrolactone/NRPS. Interestingly, comparative analysis of the biosynthetic gene clusters with S. spongiicola HNM0071^T^ revealed that S. spongiicola 531S lacked two lantipeptide gene clusters, including genes coding for the FxLD family of lantipeptides (accession no. AWK11671 to AWK11673 and AWK08909). The region of one of the two lantipeptide gene clusters in the S. spongiicola HNM0071^T^ genome was replaced by two CRISPR sequences and a gene cluster for type I-E CRISPR-associated proteins (accession no. GBP98722 to GBP98729) in the S. spongiicola 531S genome sequence.

The predicted secondary metabolite biosynthesis gene clusters of S. spongiicola 531S and their comparative analyses with S. spongiicola HNM0071^T^ will contribute to study of bioactive compounds and their biosynthetic pathways.

Streptomyces spongiicola strain 531S was deposited in the NBRC culture collection (NITE Biological Resource Center, Japan) and designated as NBRC 113560.

### Data availability.

The raw reads of Streptomyces spongiicola strain 531S have been deposited in the DDBJ Sequence Read Archive (SRA) under the accession no. DRA007446. This whole-genome shotgun sequencing project has been deposited in DDBJ/ENA/GenBank under the accession no. BGZL00000000. The version described in this paper is the first version, BGZL01000000.
